# Modeling and Simulation of a Human Knee Exoskeleton's Assistive Strategies and Interaction

**DOI:** 10.3389/fnbot.2021.620928

**Published:** 2021-03-08

**Authors:** Longbin Zhang, Yixing Liu, Ruoli Wang, Christian Smith, Elena M. Gutierrez-Farewik

**Affiliations:** ^1^KTH MoveAbility Lab, Department of Engineering Mechanics, KTH Royal Institute of Technology, Stockholm, Sweden; ^2^KTH BioMEx Center, KTH Royal Institute of Technology, Stockholm, Sweden; ^3^Department of Women's and Children's Health, Karolinska Institutet, Stockholm, Sweden; ^4^Robotics, Perception, and Learning Lab, KTH Royal Institute of Technology, Stockholm, Sweden

**Keywords:** anybody, conditional contact elements, damping factor, interactive forces, human-exoskeleton interaction

## Abstract

Exoskeletons are increasingly used in rehabilitation and daily life in patients with motor disorders after neurological injuries. In this paper, a realistic human knee exoskeleton model based on a physical system was generated, a human–machine system was created in a musculoskeletal modeling software, and human–machine interactions based on different assistive strategies were simulated. The developed human–machine system makes it possible to compute torques, muscle impulse, contact forces, and interactive forces involved in simulated movements. Assistive strategies modeled as a rotational actuator, a simple pendulum model, and a damped pendulum model were applied to the knee exoskeleton during simulated normal and fast gait. We found that the rotational actuator–based assistive controller could reduce the user's required physiological knee extensor torque and muscle impulse by a small amount, which suggests that joint rotational direction should be considered when developing an assistive strategy. Compared to the simple pendulum model, the damped pendulum model based controller made little difference during swing, but further decreased the user's required knee flexor torque during late stance. The trade-off that we identified between interaction forces and physiological torque, of which muscle impulse is the main contributor, should be considered when designing controllers for a physical exoskeleton system. Detailed information at joint and muscle levels provided in this human–machine system can contribute to the controller design optimization of assistive exoskeletons for rehabilitation and movement assistance.

## 1. Introduction

Exoskeletons have attracted increasing research interest in rehabilitation in patients with neurologic disorders, such as stroke, spinal cord injury, cerebral palsy, and Parkinson's disease (Ye et al., 2017; Fournier et al., 2018). Robotic exoskeletons are promising assistive/rehabilitative devices that can complement torque generation in people with strength deficits or assist recovery of patients with motor disorders (Yao et al., 2018; Zhang et al., 2019). Such exoskeletons can potentially alleviate therapists' intensive and tedious physical effort during rehabilitation, leaving them to focus on minimally physical interaction, observation, and supervision of patients during training (Veneman et al., 2007; Li et al., 2018b, 2019).

The mechanical design of an exoskeleton is an important factor that influences the effectiveness of its interaction with the user. Some experiments have been designed to evaluate adaptability, safety, efficiency, and comfort of an exoskeleton (Hu et al., 2016; Del Carmen Sanchez-Villamañan et al., 2019). An experimental evaluation of a passive lower extremity exoskeleton with a simple structure and a low weight was investigated with gait self-adaptivity (Wang et al., 2019), and its authors showed that the exoskeleton had the greatest influence on ankle kinematics and the least influence on hip kinematics. Li et al. (2014) evaluated an upper extremity exoskeleton with an adaptive back-stepping controller to provide assistance for the user to track predefined trajectories. Their experimental results demonstrated that the proposed adaptive controller could provide effective assistance when tracking repeated trajectories. Veneman et al. (2007) designed and evaluated a lower limb exoskeleton for interactive gait rehabilitation. Their evaluation measurements showed that the exoskeleton could follow or guide a patient, but position/angle measurement of the legs via the exoskeleton device was not sufficiently accurate for inverse dynamic calculations. While experimental evaluation of exoskeleton prototypes is important, biomechanical predictive simulations early in the design process can minimize prototype iterations and evaluate some parameters that are otherwise difficult to measure experimentally.

Most exoskeletons are evaluated with respect to their effect on the user during normal motions (Lenzi et al., 2013; Yan et al., 2015). Lenzi et al. (2013) studied the human locomotor adaptation to the action of a powered hip exoskeleton providing assistive torque with a fraction of the nominal torque profile during walking. Their experimental results showed that users could adapt and benefit from this kind of assistance by significantly reducing their muscle activation both at the hip and the ankle level. Agrawal et al. (2007) designed a passive hip and knee exoskeleton and altered the level of gravity assistance on the joints of the swing leg during walking in four able-bodied persons and three patients with stroke. They observed a number of important gait improvements in patients, such as increase in knee and hip flexion. Computational modeling of motion assistive strategies for exoskeletons has been used to analyze and estimate interaction factors on the user. The leg has been modeled as an inverted pendulum during stance and a pendulum during swing (Singh et al., 2008; Furse, 2010; Bazargan-Lari et al., 2015; Sharbafi et al., 2016). Sharbafi et al. (2017) combined a spring-mass inverted pendulum for the stance leg with a double pendulum model for the swing leg to explain human-like leg behavior in walking. The comparison between their simulations and experiments demonstrated the ability of the proposed model in replicating kinematic and kinetic behavior of both stance and swing legs. Shourijeh et al. (2017) modeled the assistive torque as a rotational actuator (RA) and applied it to hip, knee, and ankle joints. Metabolic energy was simulated with a metabolic energy rate model (Umberger, 2010) during a box-lifting task for several variations that include one to three joints. Their simulation results showed that the total metabolic energy consumption with rotational-actuator-based hip joint assistance decreased more than that with knee and ankle joint assistance. A damping component was also considered in El Zahraa Webhi et al. (2017) for an impedance control with a double pendulum model for a knee joint orthosis during swing phase. Their experimental results showed that the user's effort could be significantly decreased with this proposed approach. The exoskeletons' influences on the user at joint and muscle levels, including muscle activation, physiological joint torque, joint contact forces, and interaction forces have not been extensively described.

In simulating exoskeleton's motion assistive strategies, mass, inertia, and damping factor of its components, as well as interaction forces between exoskeletons and users, will all influence the control schemes. Dembia et al. used musculoskeletal simulations to evaluate how seven hypothetical, ideal (massless), bilateral assistive devices may affect muscle activity and metabolic cost when walking with heavy loads (Dembia et al., 2017). In their simulation results, devices that assist hip flexion, knee flexion, and hip abduction could provide greater metabolic savings than a device that assists ankle plantarflexion, and a device that assist hip abduction displayed the greatest ratio of metabolic savings to peak instantaneous positive device power. Even though they neglected the mass in their simulations, such results may guide experimental scientists in targeting joint motions. In addition, only few studies have considered the interaction forces between an exoskeleton and its user (Li et al., 2018a; Serrancoĺı et al., 2019; Su et al., 2019). Given that an exoskeleton is designed to interact directly with its wearer, it is essential to investigate the interactive forces involved. The easiest and most accurate way to determine interactive forces would be to develop prototypes and measure forces during experiments, but this would be expensive and time consuming as model design iterations are commonly needed (Cho et al., 2012). It is therefore useful when evaluating different control schemes to predict interaction forces while taking the mass, inertia, and damping factor of exoskeleton components into account.

The objectives of this study were to, in a simulation of a virtual human–machine system (HMS) with a knee exoskeleton, evaluate how different assistive strategies affect the user's required effort as well as joint contact forces, and to study the interaction forces between the device and the wearer. To illustrate these objectives, three different assistive models, namely an RA, a simple pendulum model (SPM) and a damped pendulum model (DPM), were used to model assistive torques at the knee during simulated normal and fast gait. Measurements including muscle impulse, required physiological torque, joint contact forces, and interaction forces were estimated through a computational musculoskeletal model and compared among the three assistive strategies.

## 2. Methods

**Figure 1:**
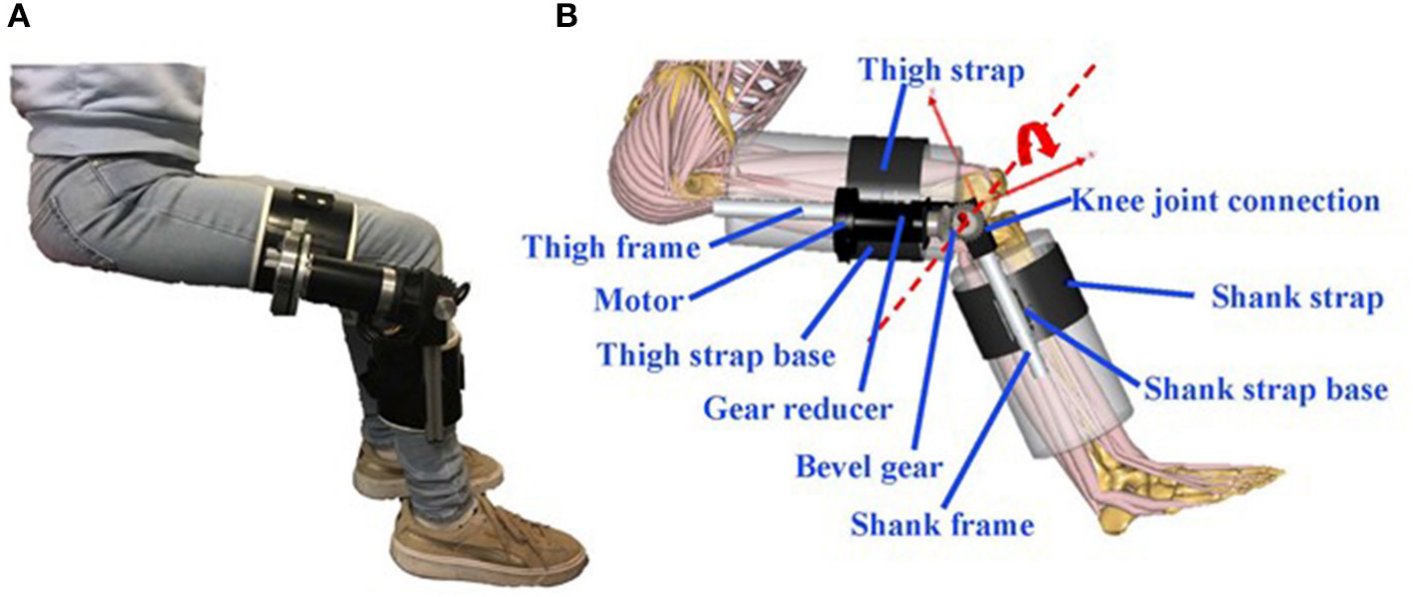
**(A)** Knee exoskeleton prototype; **(B)** virtual HMS.

In this paper, we created a human knee exoskeleton CAD model, then incorporated it into a musculoskeletal model (AnyBody AMMR), creating a virtual HMS (Figure 1). Normal and fast gait were simulated, and three different assistive torques at the knee were compared as well as a no-assistance mode. Outcome parameters were the user's required knee flexor and extensor torque, joint contact force, muscle impulse, and interaction forces in the virtual HMS.

### 2.1. Knee Exoskeleton Model

A CAD (Solidworks) model of a physical knee exoskeleton (Figure 1) was made, consisting of a Brushless DC motor (EC 90 flat ϕ90 mm 90 W; Maxon, Munich, Germany), a gear reducer (Planetary Gearhead GP 52C, Maxon, Munich, Germany), a bevel gear to change the direction of applied torque, thigh and shank cuffs, knee joint connection, and strap bases. The varied assistive torque generated by the exoskeleton was modeled as an ideal torque. Mass, moment of inertia, and damping of the exoskeleton components were incorporated into the virtual HMS.

### 2.2. Musculoskeletal Model

A musculoskeletal model was created (AnyBody Modeling System [AMS]) using the Twente Lower Extremity Model Version 2 (TLEM2.0) model. This model has been well described by Carbone et al. (2015). The mass and height of the generic model are 66 kg and 1.75 m, respectively. This model consists of one simplified upper body segment (lumbar region, rigid trunk, neck, and head) and 11 lower extremity segments: pelvis, thighs, patellas, shanks, tali, and feet. Each lower limb has four joints; the hips were modeled as three degree of freedom (DOF) ball-and-socket joints, and the knees, talocrural joints, and subtalar joints were modeled as one DOF hinges.

### 2.3. Human–Exoskeleton Interaction Model

To combine the human body model and the exoskeleton model into a virtual HMS, mechanical constraints were defined to allow their fluent interaction. The constraints between the exoskeleton and human model were modeled as a system of rigid bodies with eight DOFs: one DOF for the knee RA, one DOF for the bevel gear, three DOFs for linking the medial side of the knee, two DOFs for linking the lateral side of the knee, one DOF for linking the thigh side, and one DOF for linking the shank side. The constraints used here were all “soft” joints, which allow for small relative motions between the user skin and the exoskeleton (Damsgaard et al., 2006).

### 2.4. Knee Joint Contact Forces Computation

**Figure 2:**
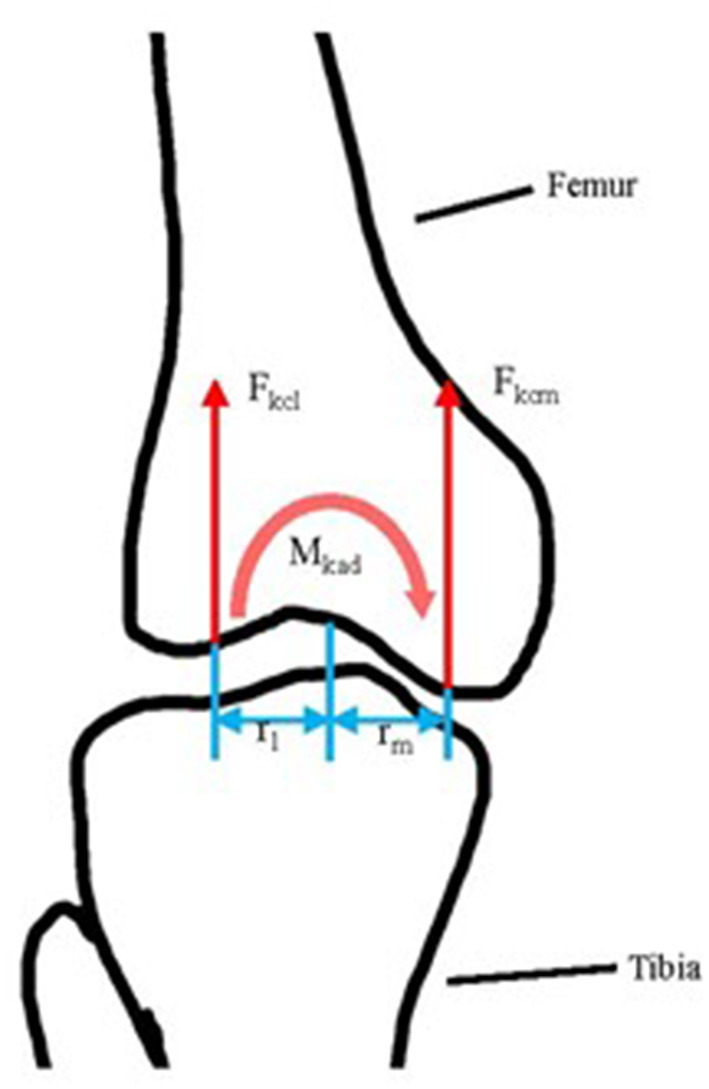
The illustration of tibiofemoral contact force computation in the frontal plane. *F*_*kcl*_ and *F*_*kcm*_: lateral and medial tibiofemoral compressive force separately; *r*_*l*_ and *r*_*m*_ are the length of the lateral and medial condyle moment arm separately; *M*_*kad*_: knee abduction moment.

Knee joint contact forces (compressive forces only) were computed as the net loading on the femur resulting from muscular forces, gravitational forces, inertial forces, and moments and ground reaction forces. The total compressive load was then decomposed into the medial and the lateral compartments by applying a moment equilibrium Equation (1) and force equilibrium Equation (2) in the frontal plane (Seedhom et al., 1972; Richards et al., 2018; Peng et al., 2020), as illustrated in Figure 2.

(1) $$\begin{array}{r} M_{kad}+F_{kcl}\cdot r_{l}-F_{kcm}\cdot r_{m}=0 \end{array}$$

(2) $$\begin{array}{r} F_{kc}=F_{kcl}+F_{kcm} \end{array}$$

where *M*_*kad*_ is the knee abduction moment in the shank coordinate system; *F*_*kcl*_ and *F*_*kcm*_ are the contact forces in the lateral and medial knee compartments, respectively; *r*_*l*_ and *r*_*m*_ are the lateral and medial condyle moment arms, respectively, and were estimated based on reported ratios of the condylar width relative to the knee width (Richards et al., 2018), and *F*_*kc*_ is the total knee contact force in the frontal plane. The contact force was normalized to body weight.

### 2.5. Interaction Forces Computation

**Figure 3:**
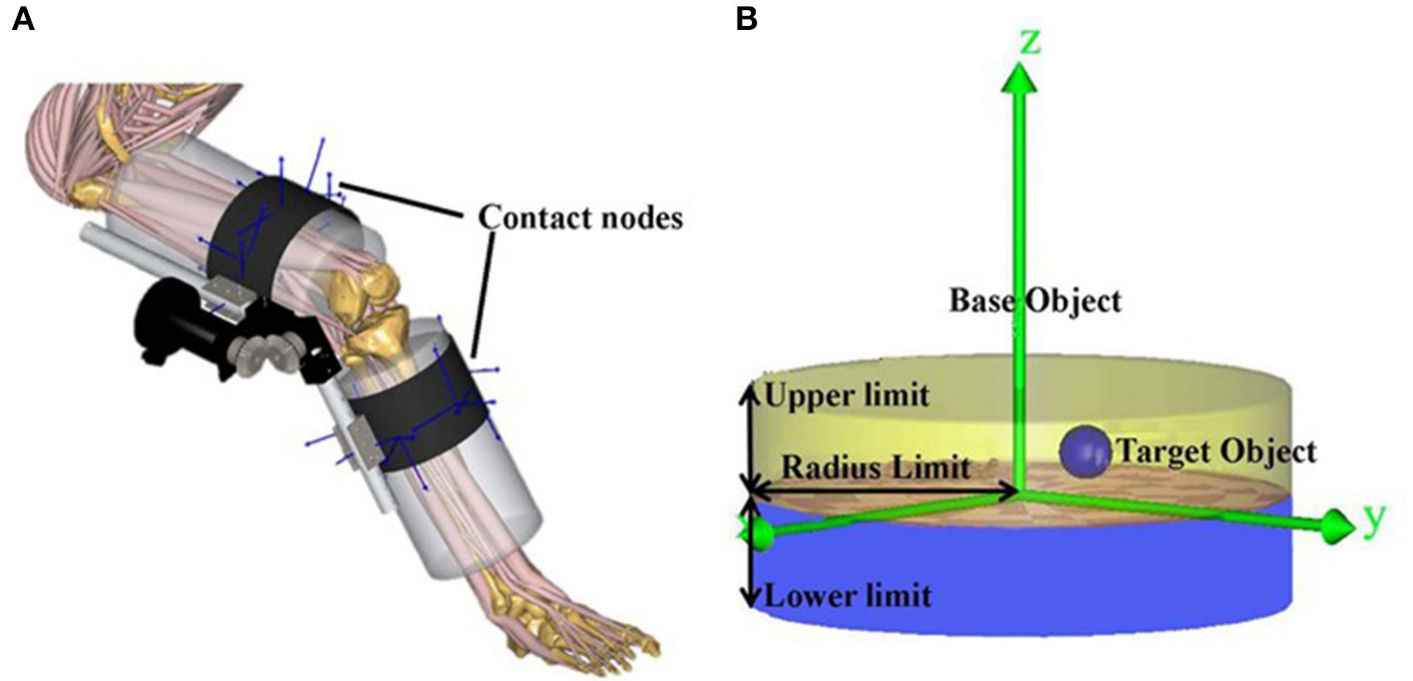
**(A)** Contact nodes between fixation straps and the user; **(B)** contact nodes were modeled as conditional contact elements between the base object (the user) and the target object (the exoskeleton). The lower and upper limits are the smallest and largest distances in the normal (*z*) direction between the base object and the target object that determines contact. The radius limit defines the distance in the tangential (*x*−*y*) plane between the base object and the target object. The modeled conditional contact elements compute the normal and tangential interactive forces between the two objects by generating artificial “muscles,” which can be recruited as any other muscles in the musculoskeletal model.

The interaction between the user and the exoskeleton fixation straps has been modeled with a number of points (Figure 3), which can transfer interactive forces from the exoskeleton to the user (Rasmussen et al., 2009). These contact nodes can provide compressive interactive (normal) forces and Coulomb tangential interactive forces proportional to the reaction forces, and were modeled as conditional contact elements in the musculoskeletal model system. Sixteen nodes on the wrapping surface of the user's modeled leg (base object) were defined, and 16 corresponding contact nodes on the exoskeleton fixation straps (target object) were defined at points of contact. Then for each contact node, a cylindrical space on the base object was defined. Contact was defined to occur when the target object was inside the virtual cylinder, and interaction forces between the two objects were computed by generating artificial “muscles.” These artificial “muscles” can be recruited as any other muscles in the musculoskeletal model and are involved in the inverse dynamics analysis.

### 2.6. Prediction of Ground Reaction Forces and Moments

The ground reaction forces and moments were predicted by creating contact elements at 25 contact nodes under each foot of the musculoskeletal model. At each contact node, five artificial muscle-like actuators were included to approximate a static Coulomb friction model; one actuator was aligned with the vertical direction of the force plate and generated a normal force, and the other two pairs of actuators were aligned with the medio-lateral and antero-posterior directions of the force plate and generated positive or negative friction forces. The computation of ground reaction forces and moments was performed in conjunction with the muscle recruitment algorithm by introducing these artificial muscle-like actuators at contact nodes, which was supported by the software (Fluit et al., 2014; Skals et al., 2017).

### 2.7. Assistive Strategies

In this study, assistive strategies were simulated during normal and fast using three models: an RA, an SPM, and a DPM. The motivations were as follows: Stiffness in an RA is proportional to joint deviation from neutral, and thus it would provide the most assistive torque in swing, since knee flexion is highest then. Furthermore, the shank acts largely like an inverted pendulum during stance and a pendulum during swing (Kuo and Donelan, 2010). Damping elements were added, as they may assist muscles' eccentric actions. The musculoskeletal simulations in this paper were based on an inverse dynamics approach. The varied assistive torque generated by the exoskeleton was modeled as an ideal torque using the “AnyForce” class.

#### 2.7.1. Rotational Actuator Model

The assistive torque modeled as an RA is formulated as Equation (3)

(3) $$\begin{array}{r} u_{r}=-K\left(\theta -\theta_{ref}\right) \end{array}$$

where *K* is the pseudo-stiffness of the assistive actuator, θ is the knee exoskeleton flexion angle, and θ_*ref*_ = 0 is the reference angle at a neutral position, i.e., a fully extended knee.

For the RA-based controller, RA stiffness has an influence on the wearer's required muscle impulse. A sensitivity analysis was performed to determine the “optimal” stiffness that induced minimal muscle impulse in the knee flexors and extensors during normal and fast gait (Supplementary Material), which we determined to be 4 *Nm*/*rad*. This value of stiffness in the RA-based controller was applied to the assistive exoskeleton.

#### 2.7.2. Pendulum Models

In the SPM and DPM, the shank-foot-lower exoskeleton (the lower half of the exoskeleton) is modeled as a common pendulum rotating about the knee for the swing leg and the shank-exoskeleton is modeled as an inverted pendulum rotating about the ankle for the stance leg.

Simple pendulum model: During swing, the dynamics of the knee modeled as a driven, simple pendulum (Ronsse et al., 2010) can be formulated with respect to time *t* as Equation (4) :

(4) $$\begin{array}{r} I_{c_{1}}{\ddot{\theta }}_{k}\left(t\right)=-m_{c_{1}}gl_{c_{1}}\sin\theta_{k}\left(t\right)+u_{s}\left(t\right) \end{array}$$

where *I*_*c*_1__ is the moment of inertia about the knee of the shank-foot-lower exoskeleton segment (computed in AMS) with the ankle in a neutral position, i.e., neglecting the inertial changes due to ankle movement, θ_*k*_(*t*) and $\ddot{\theta_{k}}\left(t\right)$ are the knee angle and angular acceleration, respectively, i.e., the shank relative to the thigh, *m*_*c*_1__ is the combined mass of the shank-foot-lower exoskeleton segments, *l*_*c*_1__ is the distance between the knee joint center and the center of mass of the shank-foot-exoskeleton pendulum, simplified to act as a rigid body during swing, *g* is the gravity constant, and *u*_*s*_(*t*) is the torque applied to the knee in the SPM-based controller.

During stance, the dynamics of the knee modeled as an inverted pendulum model (Simoneau and Corbeil, 2005; Sasagawa et al., 2014) is formulated with respect to time *t* as Equation (5):

(5) $$\begin{array}{r} I_{s}{\ddot{\theta }}_{a}\left(t\right)=T_{a}-m_{c_{2}}gl_{s}\sin\theta_{a}\left(t\right)+u_{s}\left(t\right) \end{array}$$

where *I*_*s*_ is moment of inertia about the ankle of the shank and lower exoskeleton inverted pendulum, θ_*a*_(*t*) and ${\ddot{\theta }}_{a}\left(t\right)$ are the ankle angle and angular acceleration, respectively, i.e., of the shank-exoskeleton inverted pendulum about the ankle, *m*_*c*_2__ is the combined mass of the human-exoskeleton model minus the foot segment, *l*_*s*_ is the distance between ankle joint center and the center of mass of the inverted shank-exoskeleton pendulum, and *T*_*a*_ is the ankle joint torque computed through inverse dynamics.

Damped pendulum model: During swing, the dynamics of the knee modeled as a driven, damped pendulum can be formulated as Equation (6):

(6) $$\begin{array}{r} I_{c_{1}}{\ddot{\theta }}_{k}\left(t\right)=-m_{c_{1}}gl_{c_{1}}\sin\theta_{k}\left(t\right)-b{\dot{\theta }}_{k}\left(t\right)+u_{d}\left(t\right) \end{array}$$

where *b* is the knee joint viscous damping constant, ${\overset{∙}{\theta }}_{k}\left(t\right)$ is the knee angular velocity, i.e., the shank relative to the thigh, and *u*_*d*_(*t*) is the torque applied to the knee joint in the DPM-based controller.

During stance, the dynamics of the knee modeled as an inverted pendulum can be formulated as Equation (7):

(7) $$\begin{array}{r} I_{s}{\ddot{\theta }}_{a}\left(t\right)=T_{a}-m_{c_{2}}gl_{s}\sin\theta_{a}\left(t\right)-b{\dot{\theta }}_{a}\left(t\right)+u_{d}\left(t\right) \end{array}$$

where ${\overset{∙}{\theta }}_{a}\left(t\right)$ is the angular velocity of the shank-exoskeleton inverted pendulum about the ankle.

The torque applied to the knee joint is provided by both the user and the exoskeleton. The assistive torque that is provided by exoskeleton, *u*_*es*_(*t*) for the SPM and *u*_*ed*_(*t*) for the DPM, can be expressed as a proportion *a* of the total torque applied on the knee joint during both stance and swing as: *u*_*es*_(*t*) = *a* · *u*_*s*_(*t*) and *u*_*ed*_(*t*) = *a* · *u*_*d*_(*t*); where *a* (0 ≤ *a* ≤ 1) represents the level of exoskeleton assistance. In theory, the stability limit of the assistive controller should be reached at *a* = 1. However, in reported experiments, users have expressed discomfort such as undesired high-frequency oscillations (Ronsse et al., 2011) with high levels of exoskeleton assistance. In a sensitivity analysis to determine the “optimal” assistance level *a* and damping factor *b* that induced minimal muscle impulse in the knee flexors and extensors in a gait cycle, we determined optimal assistance levels to be *a* = 0.23 during stance and *a* = 0.78 during swing for the SPM models (Supplementary Material). For the DPM models, we identified optimal values of *a* = 0.33, *b* = 12.6 for normal walking and *a* = 0.33, *b* = 18.4 for fast walking during stance, and *a* = 0.78, *b* = 0.34 for normal walking and *a* = 0.78, *b* = 0.1 for fast walking during swing.

### 2.8. Simulation Protocol

Gait at a normal velocity of 1.22*m*/*s* and at a fast velocity of 1.49*m*/*s* was simulated, using kinematics available in the software, with three different assistive modes for the exoskeleton knee joint—RA-based, SPM-based, and DPM-based assistance—as well as a no-assistance (NA) mode with added dead weight from the exoskeleton's components. Muscle impulse, required physiological knee torque, joint contact forces, and interaction forces were computed through inverse dynamic analysis, with muscle recruitment and activation resolved through static optimization with an assumption of minimum the sum of the squared muscle activation (Rasmussen et al., 2001; Damsgaard et al., 2006; Bassani et al., 2017). Muscle impulse, defined as the integral with respect to time of muscle force during one gait cycle, was computed for knee flexors: sartorius (SAR), biceps femoris long head (BFL), semitendinosus (ST), semimembranosus (SM), gracilis (GRA), gastrocnemius (GAS), and for knee extensors: vastus lateralis(VL), vastus medialis (VM), vastus intermedius (VI), and rectus femoris (RF).

## 3. Results

### 3.1. Muscle Impulse

**Figure 4:**
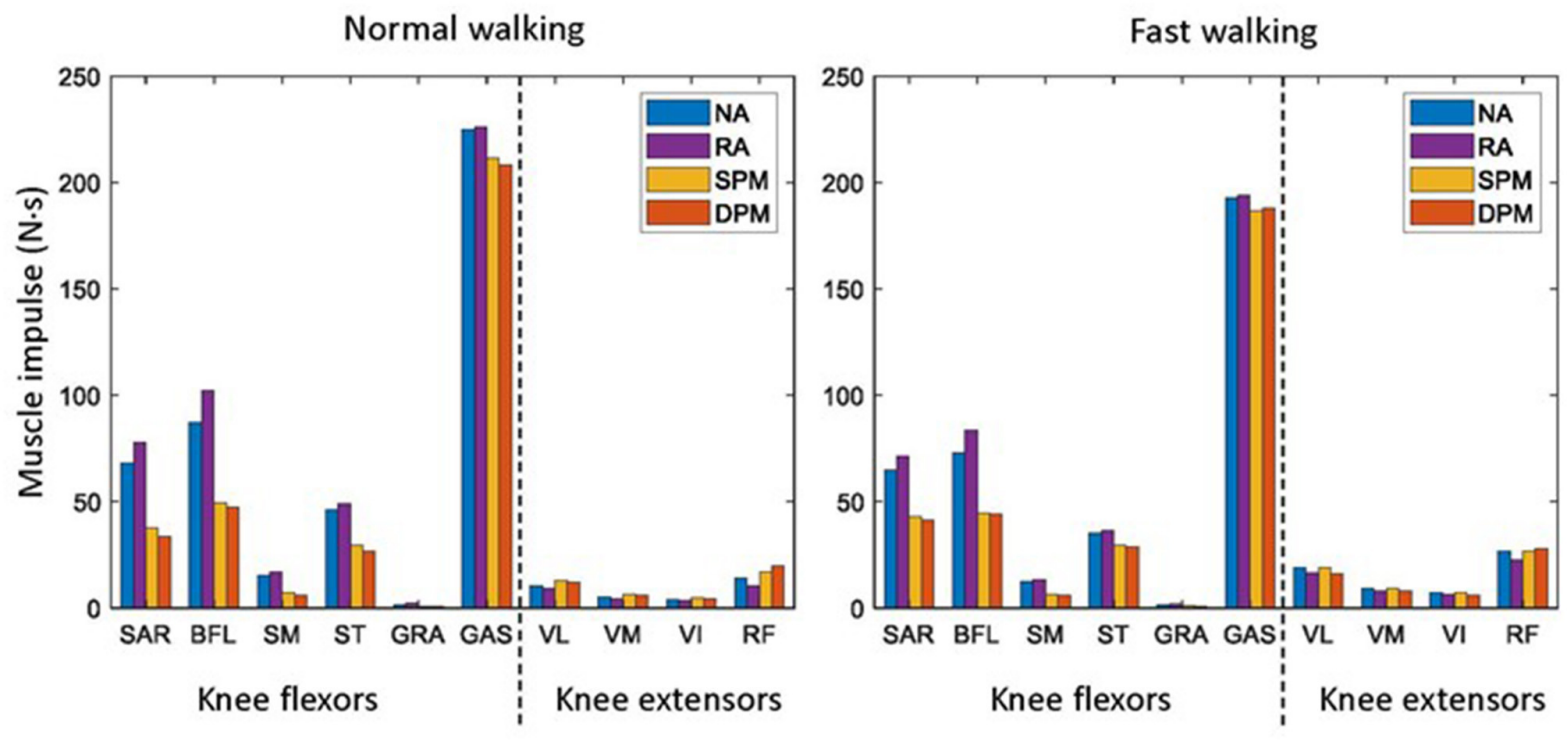
The knee flexor and extensor impulse of the user in normal **(left)** and fast **(right)** walking during a gait cycle with the exoskeleton and no assistance (NA), as well as rotational actuator (RA), simple pendulum model (SPM), and damped pendulum model (DPM) assistance modes. Knee flexors include sartorius (SAR), biceps femoris long head (BFL), semitendinosus (ST), semimembranosus (SM), gracilis (GRA), and gastrocnemius (GAS). Knee extensors include vastus lateralis (VL), vastus medialis (VM), vastus intermedius (VI), and rectus femoris (RF).

Without assistive torque from the exoskeleton (NA), muscle impulse (integral of muscle force with respect to time over one gait cycle) among knee flexors was highest in GAS followed by BFL, SAR, and ST, and was very low in GRA muscle (Figure 4). Muscle activities as time-series are illustrated in the Supplementary Material. Among the knee extensors, muscle impulse was highest in RF muscles. In the RA assistive mode, compared to NA, muscle impulse increased slightly in all knee flexors but decreased slightly in all knee flexors. In the SPM and DPM modes, among knee flexors, muscle impulse in SAR, BFL, SM, and ST decreased to approximately half that of the NA mode and decreased slightly in GAS. Compared to RA, muscle impulse in all knee extensors increased slightly in SPM and DPM modes, though the magnitudes of change were small. In DPM mode, muscle impulse was slightly lower in knee flexors and vasti muscles than that of the SPM mode but slightly higher in RF.

### 3.2. Required Physiological Knee Torque

**Figure 5:**
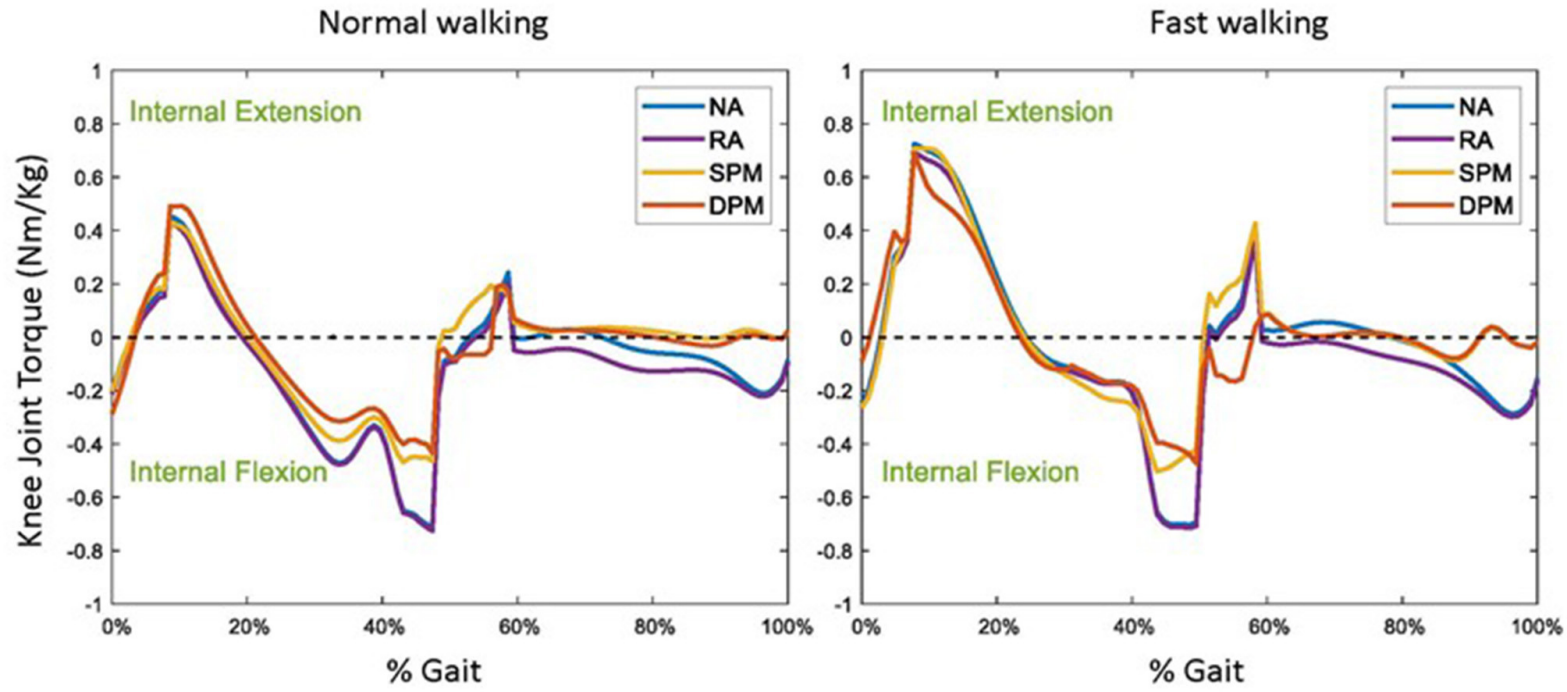
The user's required physiological knee joint torque in normal **(left)** and fast **(right)** walking, while wearing a knee exoskeleton with no assistance (NA), as well as with rotational actuator (RA), simple pendulum model (SPM), and damped pendulum model (DPM) assistive modes. Results are shown as a function of a gait cycle, 0% is initial foot contact, stance is approximately 0–60%, and swing 60–100% (Perry et al., 1992).

Without assistive torque from the exoskeleton (NA), a slight internal knee flexion torque was required in initial contact, followed by a knee extension torque in loading response, a knee flexion torque in mid-stance to late stance, a slight knee extension torque during pre-swing and a slight knee flexion torque in late swing (Figure 5). Compared to the NA mode, with the exoskeleton in RA mode, the user's required knee extension torque was slightly lower in loading response and relatively unchanged for the rest of stance, but required knee flexion torque was higher in early and mid-swing. Compared to the RA mode, with the exoskeleton in SPM and DPM modes, the required physiological knee flexion and extension torques were both lower in general. The required internal knee flexion torque decreased to approximately half that of the RA mode in the SPM and DPM modes in mid-stance and late stance and was nearly zero throughout the swing phase. At pre-swing, a slight internal knee extension torque was required with the SPM model, but a slight knee flexion torque was required with the DPM. In the swing phase, the required knee torque was nearly the same with SPM and DPM models in both normal walking and fast walking.

### 3.3. Joint Contact Forces

**Figure 6:**
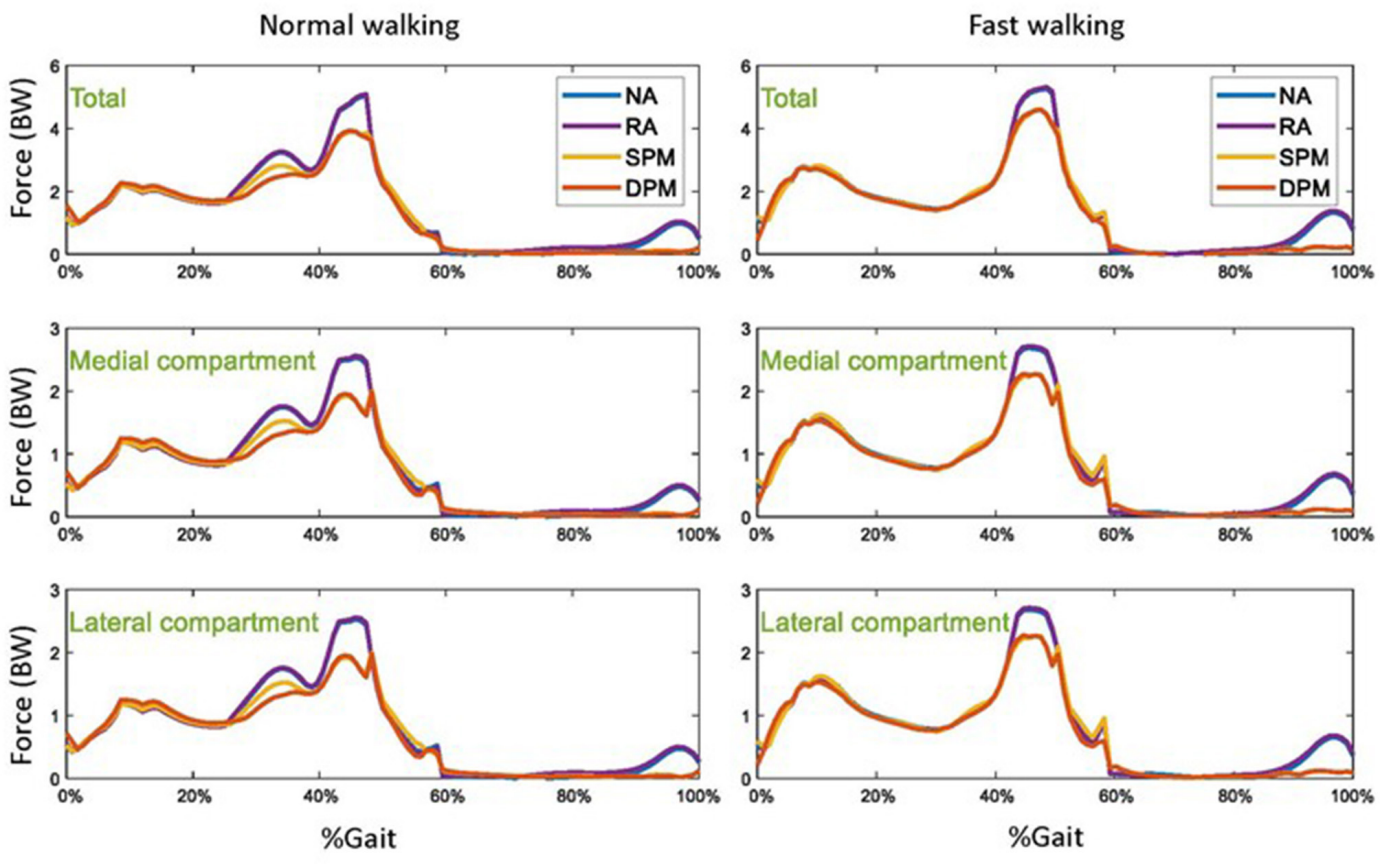
The total tibiofemoral compressive forces, and the compressive forces in the medial and lateral compartments of the knee in normal **(left)** and fast **(right)** walking, with four different modes: no assistance (NA), rotational actuator (RA) assistance, simple pendulum model (SPM) assistance, and damped pendulum model (DPM) assistance. Results are shown as a function of a gait cycle, 0% is initial foot contact, stance is approximately 0–60%, and swing is 60–100%.

Without assistive torque from the exoskeleton (NA), tibiofemoral compressive (contact) forces were approximately around 220% body weight in normal walking and 300% in fast walking in early stance, followed by around 500% body weight in normal walking and 520% in fast walking in late stance (Figure 6). Contact forces were approximately equally distribution among medial and lateral compartments. Compared to the NA mode, contact forces were practically unchanged in RA mode. In SPM and DPM modes, the tibiofemoral compressive force was lower (around 100% body weight) in late stance and was close to zero in late swing, with even distribution among compartments.

### 3.4. Interaction Forces

**Figure 7:**
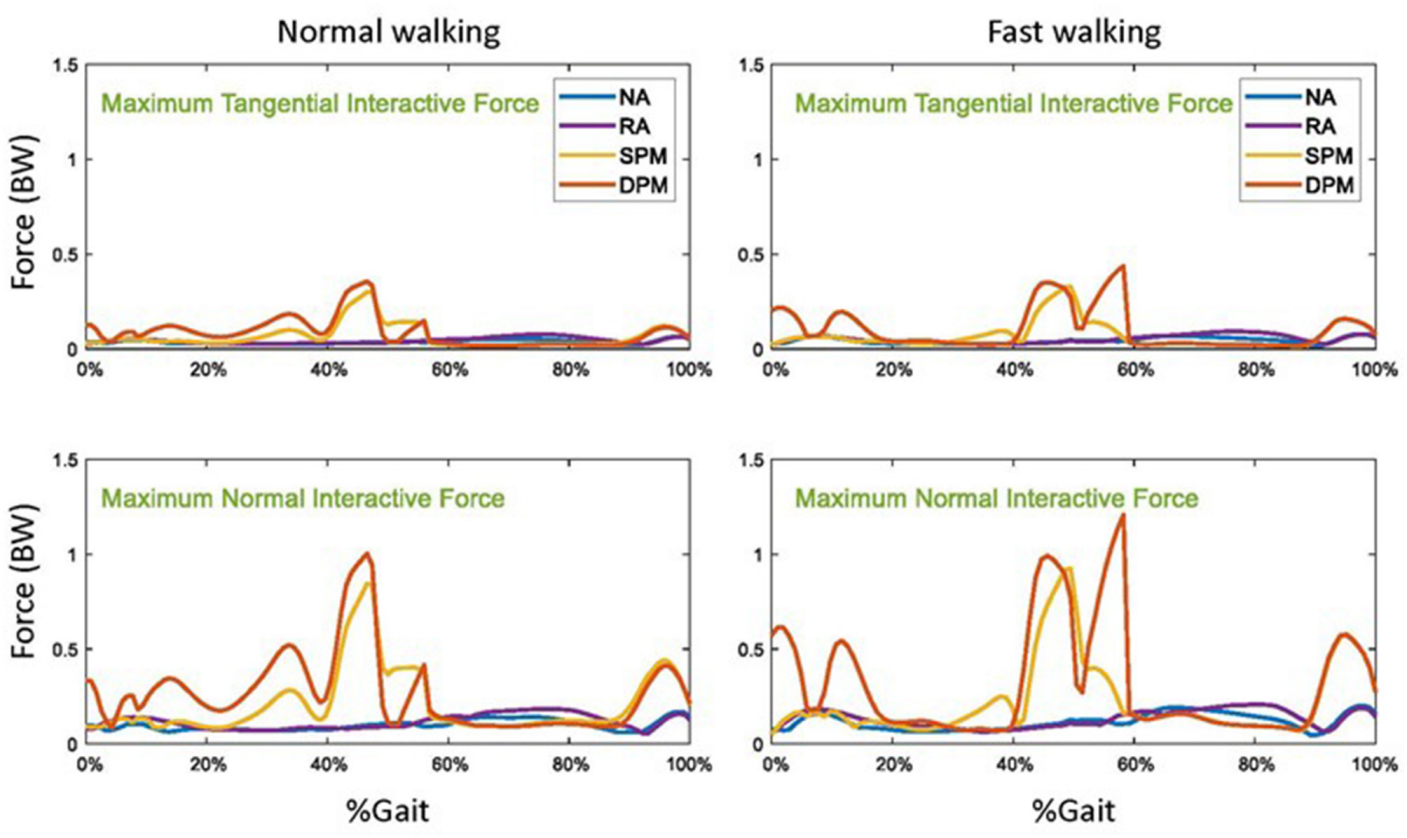
The maximum tangential and normal interactive forces between the exoskeleton straps and the user in normal **(left)** and fast **(right)** walking with no assistance (NA), as well as with rotational actuator (RA), simple pendulum model (SPM), and damped pendulum model (DPM) assistance modes. Results are shown as a function of a gait cycle, 0% is initial foot contact, stance is approximately 0–60%, and swing is 60–100%.

Without assistive torque from the exoskeleton (NA), the maximum normal and tangential interactive forces among the 16 contact nodes were highest in mid-swing but both were overall <20% body weight (Figure 7). Compared to NA, interactive forces practically unchanged in RA mode. Compared to NA mode, in SPM and DPM modes, maximal tangential interactive forces were largely unchanged in early and mid-swing, but were much higher in late stance and nearly doubled during late swing, i.e., phases in which the assistive torque compensation was highest. Similarly, compared to NA mode, maximal normal interactive force in the SPM and DPM assistive modes were practically unchanged in early and mid-swing, but were much higher in late stance and more than doubled during late swing. In SPM and DPM modes, the maximum tangential force of close to 20% body weight and the maximum normal force of over 100% body weight were seen in late stance for both walking speeds.

## 4. Discussion

We developed a virtual HMS that fully incorporates both knee exoskeleton hardware and control, and used it to compute how different assistive strategies can affect the user's required torques, muscle impulses, contact forces, and interactive forces during movement. To illustrate the potential use of the developed virtual HMS, we specifically focused on the knee in normal gait, and adapted three common but conceptually different assistive strategies at the knee joint, namely RA, SPM, and DPM strategies, to determine whether any potential benefit to the user could be identified. We found that the RA-based assistive controller could only reduce the user's required knee extensor torque and muscle impulse by a small amount. The SPM- and DPM-based assistive controllers reduced the user's required knee flexor torque and muscle impulse more than RA-based controller, with lower joint compressive contact force, but with higher interaction forces. These findings suggest that joint rotational direction should be considered, and that a potential trade-off between interaction forces and physiological torque should be considered when designing an exoskeleton controller. In addition, a damping component in a pendulum-based assistive strategy could further affect the impact on the user.

The virtual HMS we created incorporates a developed exoskeleton with a common musculoskeletal model for fidelity in using simulation to investigate human–machine interaction. While we did not target any specific patient group in this study, Figure 5 illustrates how the knee exoskeleton with the DPM-based assistive strategy could, for instance, assist a patient with weakness in the knee flexors to achieve normal and even fast walking kinematics, despite the weakness. The virtual HMS is likewise useful to explore whether other assistive strategies and/or physical designs may compensate for different muscle weakness. Virtual HMSs have been used numerous times in optimal design and control of exoskeletons. Zhou et al. (2017) created a virtual HMS by cooperating a musculoskeletal model with an upper limb exoskeleton. They evaluated two types of passive exoskeletons with gravity-compensating capability and computed optimal spring stiffness. However, different assistive strategies and the effects on the user were not studied. Pan et al. (2015) simulated a novel human–exoskeleton system by integrating ADAMS and Matlab/Simulink with fuzzy-PID/PID algorithms. They validated the efficacy of the proposed strategy in exoskeleton control to accomplish some daily activities, such as level walking, stair ascent, and squatting down. However, they did not compute the user's effort and interactive forces either. Simulation-based exoskeleton and assistive strategy design has become more common in recent years. A recent work by Shourijeh et al. (2017) describes assistive exoskeleton torques applied to the hip, knee or ankle joint modeled as RAs, and reports their effects on the overall metabolic energy expenditure. Carmichael and Liu (2011) used a musculoskeletal model to estimate the strength of the user and concluded that such information could be useful in improving the control schemes for robots that physically assist people. Agarwal et al. (2011) analyzed the performance of an elbow exoskeleton in four different case scenarios, and reported the effects on muscle forces and elbow torques of the user. De Rossi et al. (2011), in an experimental study, measured the pressure distribution in a user wearing a lower limb exoskeleton, using an array of soft silicone pressure sensors. This type of study is clearly useful in assessing the safety and comfort of human–machine interactions, but methodological limitations in experimental studies make it impossible to study other important information in the human–machine interaction, such as joint contact force and the effects of different assistive strategies. Using the virtual HMS in the current study, detailed information at joint and muscle levels as well as interaction forces were investigated and analyzed. Our proposed virtual HMS thus addresses several knowledge gaps in published literature, particularly in simulation-based studies.

In the illustrative study of the knee joint during gait, we found that, compared to no assistance, assistance torque based on pendulum models can reduce the demand on the user's knee flexors while the RA-based model reduced the demand on knee extensors. As the types of assistance are conceptually different, they affected the user's required muscle effort in different ways. In initial contact, late stance and late swing, the RA-based assistance did not complement the user's required knee flexion torque; the knee extensors' muscle impulse decreased but the knee flexors' impulse increased. This is related to the design and parameters in Equation (3); the RA assistance provides torque proportional to the knee angle's deviation from full extension. During gait, maximum knee flexion occurs in early to mid-swing, but only a small amount of knee extension torque is required. Therefore, the RA-based model reduced the demand on the user's knee extensors by a small amount but increased the knee flexors' impulse. The RA assistive torque, however, does not account for motion direction at the knee, and thus providing knee extension assistance and obstructing the increasing flexion of the knee in early swing. The RA-based model even continued to provide knee extension assistance in mid-swing to late swing, whereas knee flexion torque was required to slow the rate of knee extension. During initial contact, late stance, and late swing, the knee is nearly in full extension, and the assistive torque was therefore low. This illustrates that an assistive strategy should consider the motion direction, not just the position deviation from a reference value, of the joint it assists, such as the pendulum models. In the illustrative study, assistance based on the SPM or the DPM resulted in lower demand on the user's knee flexors than the RA model, but also in higher interaction forces. The lower knee flexor effort was the result of the shank-foot-lower exoskeleton acting much like a rigid pendulum rotating about the knee in mid-swing to late swing, and its inertia was in this case beneficial. The addition of the damping component in pendulum-based assistance made little difference during swing, but further decreased the user's knee flexion torque during late stance when the knee stiffens to stabilize the upper body and may benefit from a higher damping coefficient (Zhao et al., 2013).

Despite the reduced demand on the user's muscles with the SPM and DPM modes, it is notable that the interactive forces between the user and exoskeleton straps were higher in these pendulum-based models in late stance and late swing in the example study. These higher interaction forces might be expected to cause discomfort, pain, or skin damage to the user. This trade off should be take into account when designing a controller for a physical system. When an exoskeleton transmits assistive torque to the user, interactive forces at attachment locations may result; when assistive torque is highest, the interactive forces can likewise be expected to be highest. The predicted interactive forces were computed by generating artificial “muscles” in the computational musculoskeletal model. These artificial “muscles” can be recruited as any other muscles in the musculoskeletal model and are involved in the inverse dynamics analysis. Olesen et al. (2014) applied a similar method to compute the forces between the user and a chair at different seated postures. Compared predicted with measured interactive forces, they found that the predicted and measured interactive forces had similar trends in different seated postures, and could thus be used as important indicators of how different seated postures would affect the user. Thus, the interactive forces in the proposed virtual HMS could be an important indicator of the user's comfort.

We found highest compressive tibiofemoral force as well as in sartorius and hamstrings impulse during late stance and late swing in the illustrative study with no assistive mode. The greatly reduced compressive tibiofemoral force in the SPM and DPM modes can be in part attributed to the reduced muscle impulse during late stance and late swing. Compressive tibiofemoral force has frequently been used as an indicator in gait analysis (Lee and Wang, 2015; Wang et al., 2016; Mannisi et al., 2019), wherein larger tibiofemoral force may result in knee pain. Pizzolato et al. (2017) estimated the tibiofemoral contact force using an electromyography-driven neuromusculoskeletal model in real time, and used it as a visual biofeedback variable for gait modification. Lee and Wang (2015) reported on a design concept of relieving compressive contact force in the knee to obtain optimal designing parameters with a passive lower extremity exoskeleton. The ability to estimate tibiofemoral force during gait analysis therefore has potential use in rehabilitation and therapy.

There are some limitations in this study. Three relatively simple and passive assistive strategies were simulated in this virtual HMS. The shank-foot-lower exoskeleton was simplified to act as a single pendulum during swing, rather than as a double pendulum; the relative movement of the foot with respect to the shank during swing might result in a small error between the estimated knee joint torque and the actual knee joint torque. Also, when modeling the interactive forces, the strap's tension, material, and surface friction were not considered, nor were the soft tissue properties of the user's thigh and shank. Instead, they were modeled with a number of points at which normal and tangential interactive forces can be transferred between the exoskeleton and the user. As such, the trends of interactive forces, rather than the magnitudes, should be considered. Finally, the difficulty of validating computational methods that compute joint contact forces (Jung et al., 2017) and interactive forces (Olesen, 2008) are well-documented. The aim of this study, to evaluate through simulation of an HMS how different assistive strategies may affect the user, should thus be considered in the context of these limitations.

## 5. Conclusion

In this paper, we created a virtual HMS that fully incorporates both a knee exoskeleton hardware and a musculoskeletal model. This proposed virtual HMS in general can be useful for simulating different assistive strategies, as well as for analyzing how these different strategies can affect a number of parameters that describe the demands and consequences on the user, including muscular demand, joint contact forces, and human–machine interactive forces involved in movements. Detailed information at joint and muscle levels provided by using a musculoskeletal modeling environment can contribute to optimal controller design of exoskeletons.

The results of the illustrative comparison of three conceptually different assistive strategies at the knee during gait suggest that both joint position and rotational direction should be considered in an assistive strategy, and a damping component in a pendulum-based assistive strategy could be beneficial to further reduce the user's required muscle effort during the stance phase. We have also demonstrated that a higher assistive torque may incur a trade-off in the form of higher interaction forces, which should be kept in mind when designing a control strategy.

## Data Availability Statement

Publicly available datasets were analyzed in this study. This data can be found at: https://anyscript.org/ammr-doc/auto_examples/Mocap/plot_Plug-in-gait_Simple_LowerExtremity.html#sphx-glr-auto-examples-mocap-plot-plug-in-gait-simple-lowerextremity-py.

## Author Contributions

YL developed the knee exoskeleton CAD model. LZ incorporated the CAD model into a musculoskeletal model, designed the particular research question, performed the simulations, and drafted the manuscript. EG-F and RW supervised the work progress and helped draft and revise the article. CS supervised the work progress. All authors contributed to the article and approved the submitted version.

## Conflict of Interest

The authors declare that the research was conducted in the absence of any commercial or financial relationships that could be construed as a potential conflict of interest.
