# Supplementary material for: Modeling and Simulation of a Human Knee Exoskeleton's Assistive Strategies and Interaction
Source: Front Neurorobot. 2021 Mar 8;15:620928. doi: 10.3389/fnbot.2021.620928 (PMC7982590; doi:10.3389/fnbot.2021.620928)
Supplement: Supplementary file 1 [file Data_Sheet_1.PDF]

Supplementary Material

1 PARAMETER STUDY OF STIFFNESS IN RA

We performed a sensitivity analysis of how a range of K pseudo-stiffness values of in the RA-based model influences the user's muscle impulse, by computing individual muscle forces through inverse dynamic analysis and static optimization. We identified an optimal stiffness of $K = 4$ Nm/rad (Fig. S1) at which knee muscle impulse was lowest in normal and fast walking. Muscle impulse was computed as integrated muscle force over a gait cycle. The muscle force was computed in knee flexors: sartorius (SAR) biceps femoris long head (BFL), semitendinosus (ST), semimembranosus (SM), gracilis (GRA), gastrocnemius (GAS); and extensors: vastus lateralis (VL), vastus medialis (VM), vastus intermedius (VI), rectus femoris (RF).

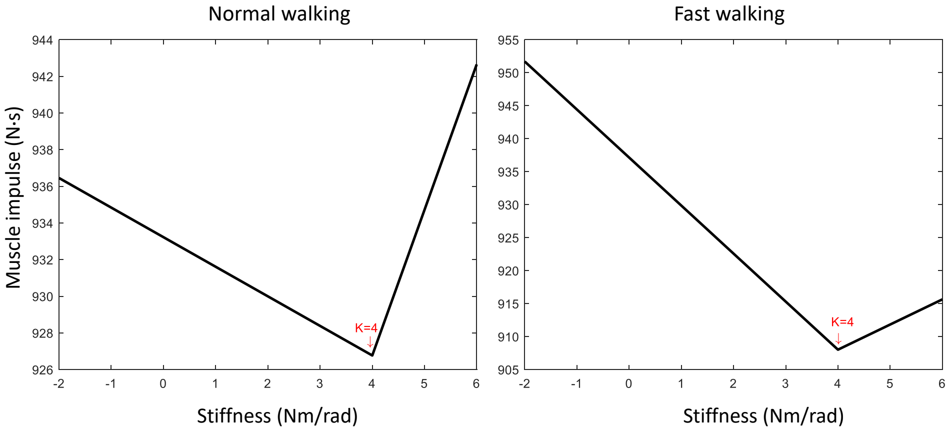

Figure S1. The knee flexor and extensor impulse of the user in normal and fast walking over the entire gait cycle, with the RA-based controllers set at a range of stiffnesses K. The lowest muscle impulse was found at a stiffness value of $K = 4$ Nm/rad for both walking speeds.

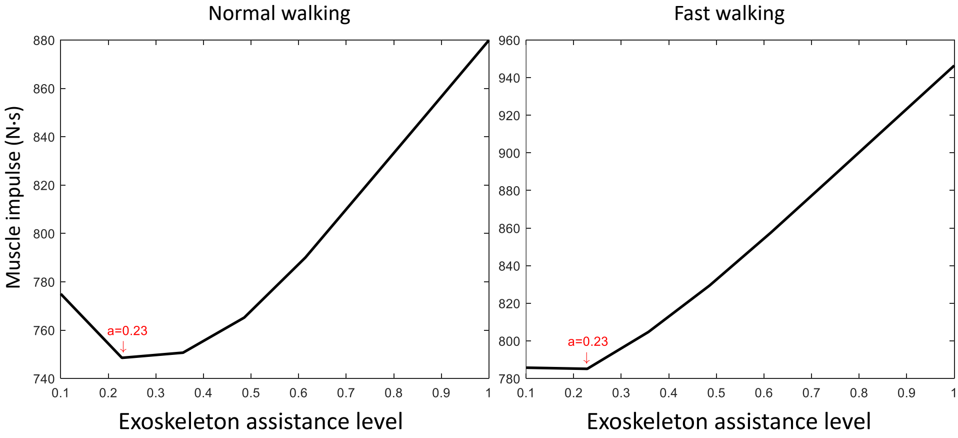

Figure S2. The knee flexor and extensor impulse of the user in normal and fast walking during the stance phase, with the SPM-based controllers set at a range of exoskeleton assistance level a $(0 \leq a \leq 1)$. The lowest muscle impulse was found at a proposition value of $a = 0.23$.

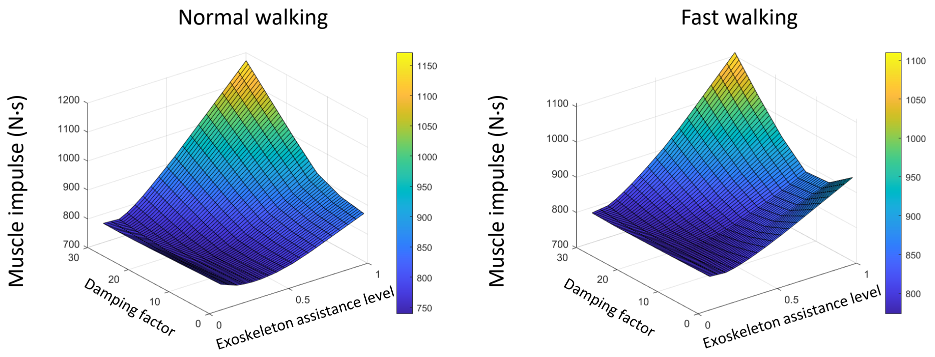

Figure S3. The knee flexor and extensor impulse of the user in normal and fast walking during the stance phase, with the DPM-based controllers set at a range of exoskeleton assistance level a $(0 \leq a \leq 1)$. The lowest muscle impulse was found at a proposition value of $a = 0.33, b = 12.6$ for normal walking and $a = 0.33, b = 18.4$ for fast walking.

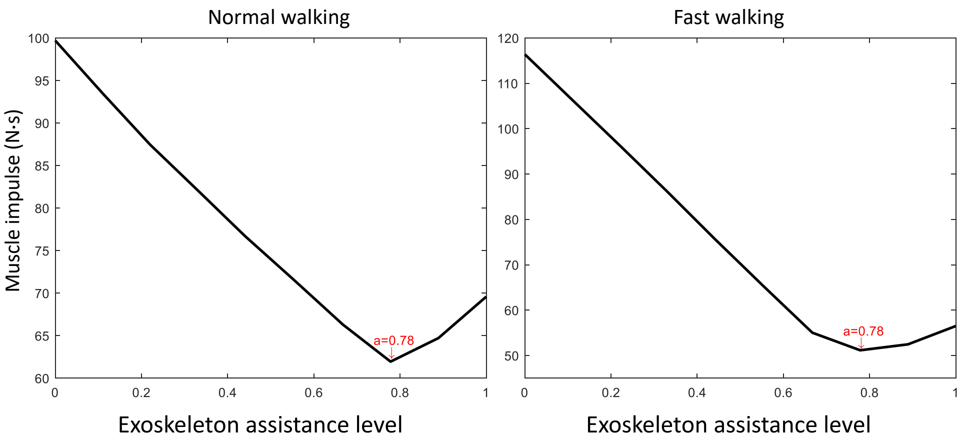

Figure S4. The knee flexor and extensor impulse of the user in normal and fast walking during the swing phase, with the SPM-based controllers set at a range of exoskeleton assistance level a $(0 \leq a \leq 1)$. The lowest muscle impulse was found at a proposition value of $a = 0.78$.

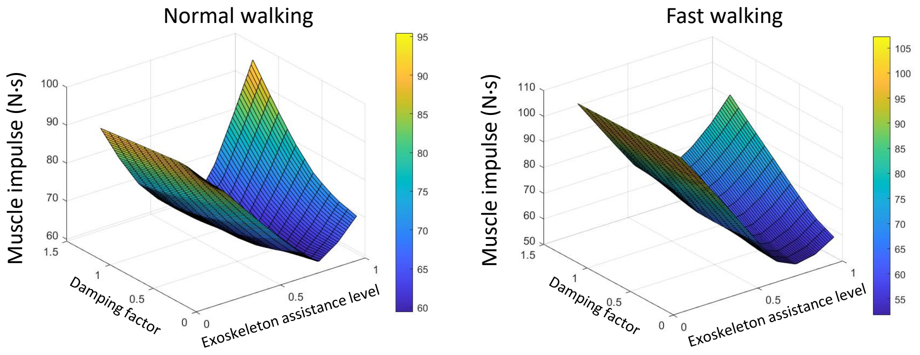

Figure S5. The knee flexor and extensor impulse of the user in normal and fast walking during the swing phase, with the DPM-based controllers set at a range of exoskeleton assistance level a $(0 \leq a \leq 1)$. The lowest muscle impulse was found at a proposition value of $a = 0.78, b = 0.34$ for normal walking and $a = 0.78, b = 0.1$ for fast walking.

2 SENSITIVITY STUDY OF EXOSKELETON ASSISTANCE LEVEL'S INFLUENCE ON MUSCLE IMPULSE IN SPM AND DPM

We performed two separate parameter studies of how a range of exoskeleton assistance level a and damping factor b values of in the SPM- and DPM-based models influences the user's muscle impulse, one for the inverted pendulum with respect to the stance leg and one for the common pendulum with respect to the swing leg. We identified optimal values of $a = 0.23$ during the stance phase and $a = 0.78$ during the swing phase for the SPM models at which knee muscle impulse was lowest in normal and fast walking (Fig. S2 and Fig. S4). For the DPM models, we identified optimal values of $a = 0.33, b = 12.6$ in normal walking and $a = 0.33, b = 18.4$ in fast walking during the stance phase, and $a = 0.78, b = 0.34$ in normal walking and $a = 0.78, b = 0.1$ in fast walking during the swing phase (Fig. S3 and Fig. S5).

3 MUSCLE ACTIVATION IN TIME-SERIES IN NORMAL AND FAST WALKING

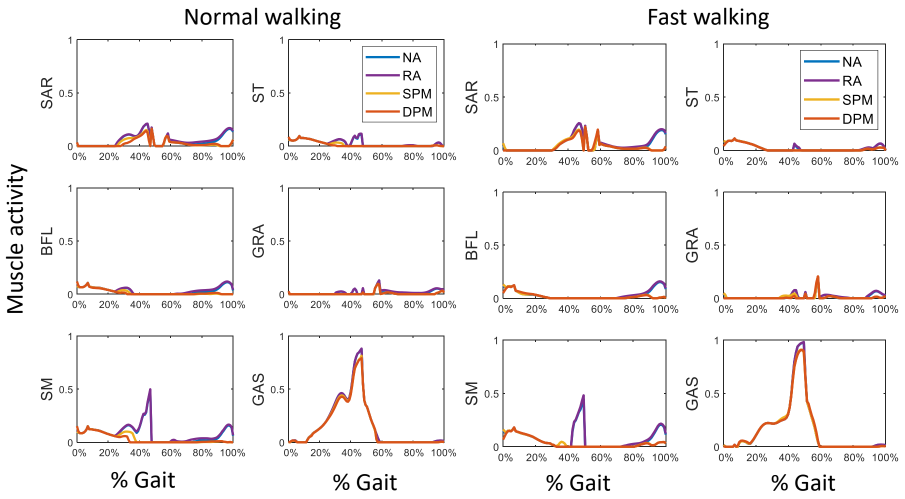

Figure S6. The knee flexor muscle activity of the user in normal and fast walking with the exoskeleton and no assistance (NA), as well as rotational actuator (RA) assistance, simple pendulum model (SPM) assistance and damped pendulum model (DPM) assistance. Knee flexors include sartorius (SAR), biceps femoris long head (BFL), semitendinosus (ST), semimembranosus (SM), gracilis (GRA), gastrocnemius (GAS).

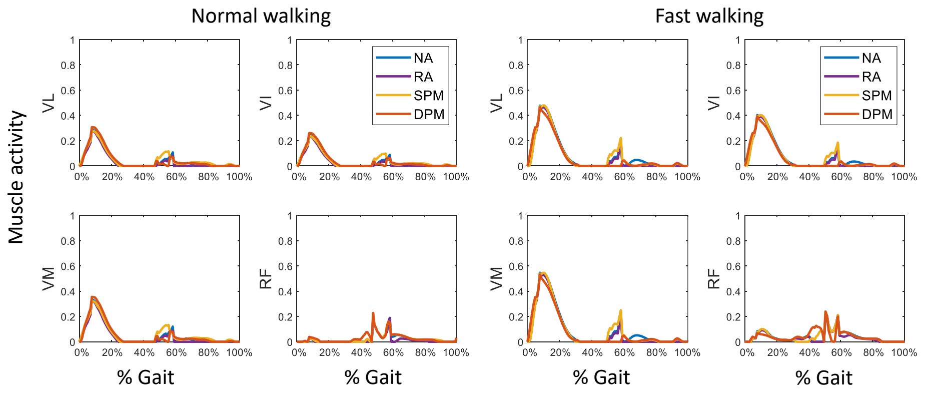

Figure S7. The knee extensor muscle activity of the user in normal and fast walking with the exoskeleton and no assistance (NA), as well as rotational actuator (RA) assistance, simple pendulum model (SPM) assistance and damped pendulum model (DPM) assistance. Knee extensors include vastus lateralis (VL), vastus medialis (VM), vastus intermedius (VI), rectus femoris (RF).
